# The yields of nucleic acids and proteins extracted from various murine tissue types

**DOI:** 10.3389/abp.2026.16122

**Published:** 2026-07-29

**Authors:** Yifan Chen, Xixuan Dong, Lixiang Xue, Zhongnan Yin

**Affiliations:** 1 Institute of Medical Innovation and Research, Peking University Third Hospital, Beijing, China; 2 Cancer Center, Peking University Third Hospital, Beijing, China; 3 Biobank, Peking University Third Hospital, Beijing, China; 4 National Human Genetic Resources Center, National Research Institute for Family Planning, Beijing, China

**Keywords:** biobank, DNA yield, mouse tissue samples, protein yield, RNA yield

## Abstract

**Objective:**

To characterize deoxyribonucleic acid (DNA), ribonucleic acid (RNA), and protein yields extracted from tissue of fourteen mouse organ types to establish baseline yield estimates for these biomolecules and provide guidance for tissue sample usage and storage in biomedical studies.

**Methods:**

Six wild-type C57BL/6J and six BALB/c nude mice were euthanized and fresh tissues were collected for extracting total DNA, RNA, and protein. The concentration, purity, and quality of nucleic acids were measured with NanoDrop™ One spectrophotometer, gel electrophoresis or capillary electrophoresis. Protein concentration was determined using the bicinchoninic acid (BCA) method.

**Results:**

DNA yields were highest in the colorectum and lowest in bone. RNA yields were highest in the colorectum and spleen of C57BL/6J and BALB/c nude mice, respectively, and lowest in bone. Protein yields were highest in the kidney and liver of C57BL/6J and BALB/c nude mice, respectively, and lowest in bone. The tissue quantities required to obtain 10 μg of DNA, 10 μg of RNA, and 1 mg of protein were calculated and presented along with corresponding tissue sizes.

**Conclusion:**

DNA, RNA, and protein yields varied significantly across different mouse tissue types. These findings provide a reference for estimating tissue sample sizes needed for downstream assays and optimizing the preservation of mouse tissues.

## Introduction

DNA, RNA, and proteins are fundamental to biomedical research. Advances in high-throughput technologies, such as whole-genome sequencing, RNA sequencing, and metabolomics, have increased the demand for high-quality tissue samples (Bagger et al., 2024; Zhao et al., 2023; Eldjarn et al., 2023; Smail and Montgomery, 2024; Want et al., 2012). These approaches enable molecular profiling of diseases and facilitate the discovery of biomarkers and disease mechanisms (Kalia, 2015; Ross, 2011). Appropriate preservation of tissue samples, especially those that are rare or difficult to acquire, is essential to maximize their research value (Yu and Zhu, 2010).

Often, the same original sample is allocated for multiple assays, requiring aliquoting into smaller portions for different types of analyses. This poses a challenge for biobanks, where storage space is limited. Thus, it is critical to determine optimal sample amounts that ensure sufficient material for future studies while conserving storage resources. However, systematic data on DNA, RNA, and protein yields from fresh tissues are lacking. Previous studies have reported yields from autopsy-derived or formalin-fixed tissues (Pearce et al., 2024; Austin et al., 2016; Siuta et al., 2023; Stroh et al., 2021), but long-term storage or chemical treatment can degrade nucleic acids and proteins, making those estimates inapplicable to fresh or short-term cryopreserved tissues. Certain applications, such as metabolic profiling or DNA methylation analysis, specifically require fresh or non-formalin-fixed tissues (Dumenil et al., 2014; Want et al., 2012). Previous studies have demonstrated that nucleic acid and protein yields vary across tissue types and mouse strains. For instance, plasma protein concentrations differ substantially between C57BL/6J and BALB/cJ mice (Michaud et al., 2018), and protocols for RNA extraction from challenging tissues such as bone have been established using column-based methods (Marek et al., 2019). Commercial technologies now enable simultaneous stabilization and purification of DNA, RNA, and protein from a single tissue sample, underscoring the feasibility of multi-analyte yield assessments. However, a systematic baseline for DNA, RNA, and protein yields across fresh tissues from multiple murine strains remains lacking.

In this study, we quantified DNA, RNA, and protein yields from fresh tissues of fourteen organ types across two mouse strains. Our results provide baseline yield values for these biomolecules, facilitating better planning of tissue storage and use in downstream applications.

## Materials and methods

### Animals

Female wild-type C57BL/6J mice (9–10 weeks old, 18–22 g) and BALB/c nude mice (7–8 weeks old, 18–20 g) were obtained from the Animal Center of Peking University Health Science Center. All procedures were approved by the Institutional Animal Care and Use Committee of Peking University (PUIRB-LA2023330). All the methods and experimentation were performed according to the relevant regulations and guidelines, including the ARRIVE guidelines. Mice were housed under standard conditions with *ad libitum* access to food and water. After a two-day acclimatization period, mice were euthanized through cervical dislocation, and the following tissues were collected: bone (from the tibia and femur excluding bone marrow), colorectum, heart, kidney, liver, lung, lymph node (from the inguinal and axillary regions), muscle (from the gastrocnemius and tibialis anterior muscles of the leg), ovary, skin, spleen, stomach, thymus, and uterus from C57BL/6J mice; since the thymus and lymph nodes are absent or minimally developed in BALB/c nude mice, only the other twelve types of tissue were collected. Cervical dislocation was performed without prior anesthesia, as the use of anesthetics may interfere with downstream molecular analyses; this method is accepted for rapid euthanasia by trained personnel (AVMA Guidelines for the Euthanasia of Animals: 2020 Edition). Tissue weights ranged from 6 to 45 mg. Larger tissues were divided into small pieces and homogenized using a Mixer Mill (Retsch, 30 Hz, 3 min) in appropriate lysis buffer until no visible lumps remained.

### Extraction of DNA, RNA, and protein

DNA and RNA were extracted from separate tissue aliquots using the QIAamp DNA Mini Kit and RNeasy Plus Universal Mini Kit (Qiagen), respectively. All procedures were performed strictly in accordance with the manufacturer’s instructions using manual procedures performed individually per analyte. Elution volumes were 50 μL for DNA and 40 μL for RNA. Concentration and purity (260/280 nm ratio) were determined using a NanoDrop™ One spectrophotometer. DNA integrity was evaluated by agarose gel electrophoresis; approximately 1 μg of genomic DNA was mixed with 2 μL of 0.25% bromophenol blue solution and separated at 16 V/cm for 30 min on a 1% agarose gel in Tris-borate-EDTA (TBE) buffer. DNA bands were visualized under ultraviolet (UV) light and photographed using the Tanon System. RNA quality was assessed using capillary electrophoresis (Fragment Analyzer 5200).

For protein extraction, tissues (except bone) were homogenized in radioimmunoprecipitation assay (RIPA) buffer (Thermo Scientific, 25 mM Tris-HCl pH 7.6, 150 mM NaCl, 1% NP-40, 1% sodium deoxycholate, 0.1% sodium dodecyl sulfate (SDS) supplemented with Protease Inhibitor Cocktail (AMRESCO), incubated on ice for 30 min, and centrifuged at 13,600 g for 20 min at 4 °C. The supernatant was collected as total protein, as previously reported (Chen et al., 2025). Bone tissue was processed using a Bone Tissue Protein Extraction Kit (Aidisheng): Fresh bone tissue samples were soaked in phosphate-buffered saline (PBS) buffer at 4 °C twice. The bones were then washed with distilled water to remove any residual red blood cells. The bone tissue was cut into small pieces, weighed, and placed into a mortar filled with liquid nitrogen, where it was ground into a fine powder. 500 μL of Protein Extract A, along with 2 μL of protease inhibitor and 2 μL of protein stabilizer, was added to 200 mg of bone tissue. The mixture was shaken at 4 °C for 30 min and subjected to ultrasound at 80–100 W for 20 cycles in an ice bath (5s ultrasound/5s interval). Subsequently, the mixture was centrifuged at 4 °C at 12,000 g for 10 min. The supernatant was collected as the total protein extract from the bone tissue. Protein concentrations were measured using the bicinchoninic acid (BCA) Protein Assay kit (Thermo Scientific). A total of 25 μL of protein sample and 200 μL of BCA working solution were added to a 96-well plate, followed by incubation at 37 °C for 30 min. The absorbance was then measured at 570 nm using a microplate reader (Tecan). A standard curve (y = 1.2416x - 0.1685, R^2^ = 0.996) was generated based on the absorbance values of various concentrations of bovine serum protein standards (0 μg/mL, 25 μg/mL, 125 μg/mL, 250 μg/mL, 500 μg/mL, 750 μg/mL, 1,000 μg/mL, 1,500 μg/mL, and 2000 μg/mL).

### Statistical analysis

For each tissue type, six biological replicates (n = 6 per strain) were used. Data are now presented as mean ± standard deviation. Data were analyzed using GraphPad Prism 9.3.0. One-way ANOVA with *post hoc* tests (Newman-Keuls, Tukey, or Kruskal-Wallis) was used for multiple comparisons. A p-value <0.05 was considered significant.

## Results

### Purity of DNA extracted from tissues of different organ types

**FIGURE 1:**
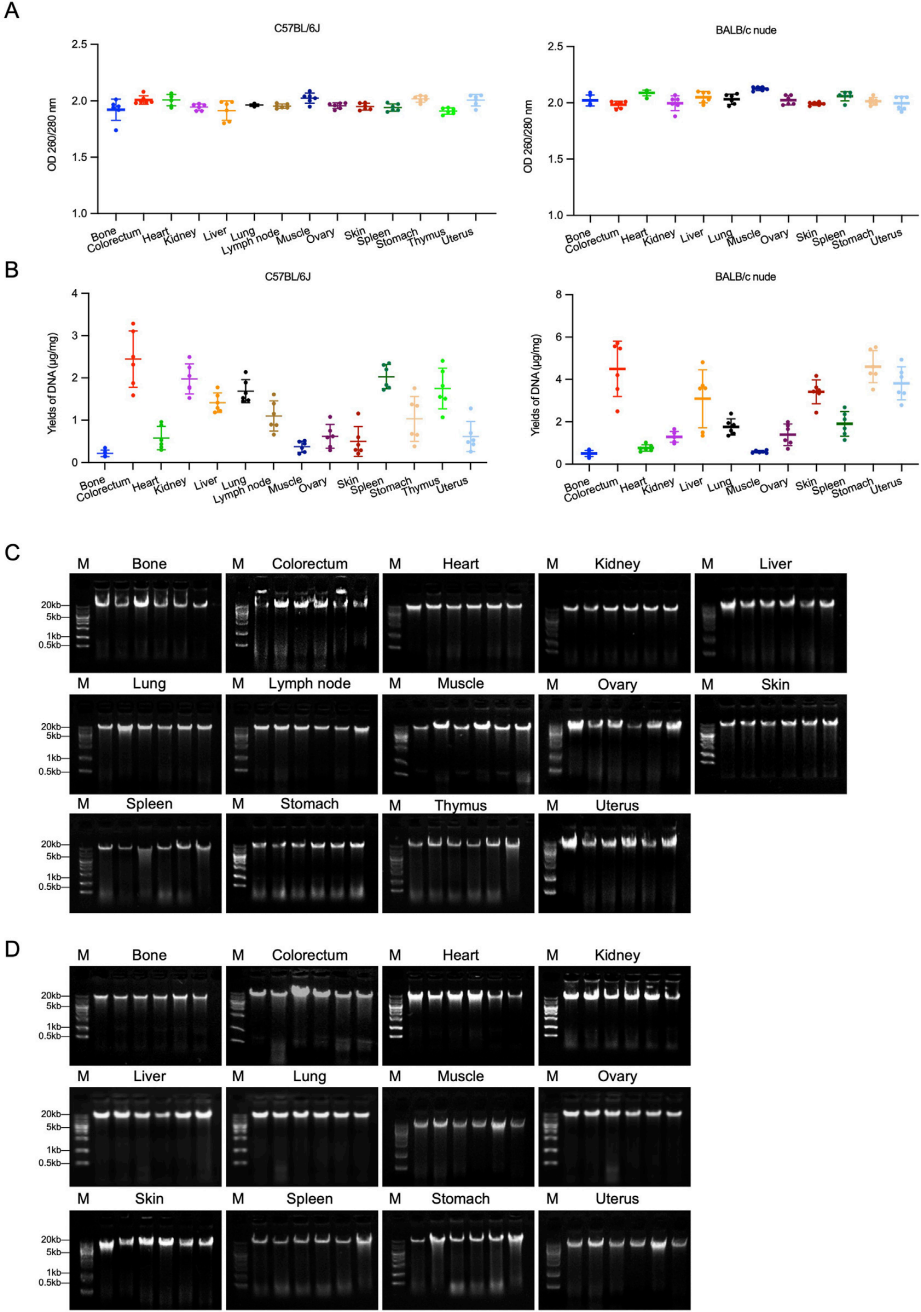
Purity, yields, and quality of DNA extracted from tissue of different types of organs. **(A)** The ratios of OD 260 nm to OD 280 nm of the DNA extracted from bone, colorectum, heart, kidney, liver, lung, lymph node, muscle, ovary, skin, spleen, stomach, thymus, and uterus in C57BL/6J (fourteen organ types) and BALB/c nude mice (twelve organ types). The results were shown as boxplots, n = 6. **(B)** Yields of the DNA extracted from tissue of twelve (BALB/c nude) to fourteen (C57BL/6J) organ types. The results were shown as boxplots (n = 6). **(C)** Gel electrophoresis of the extracted DNA from C57BL/6J mice. **(D)** Gel electrophoresis of the extracted DNA from BALB/c nude mice. M: DNA marker.

DNA was extracted from mouse tissues of various organs, including bone, colorectum, heart, kidney, liver, lung, lymph node, muscle, ovary, skin, spleen, stomach, thymus, and uterus. The optical density (OD) ratios at 260/280 nm of DNA from each group were measured using a NanoDrop™ One microvolume spectrophotometer (Figure 1A). In C57BL/6J mice, the average 260/280 ratios across the fourteen tissue groups ranged from 1.91 ± 0.03 to 2.02 ± 0.04, with no statistically significant differences among groups (P > 0.05). Similarly, in BALB/c nude mice, the average ratios across twelve groups ranged from 2.00 ± 0.06 to 2.13 ± 0.02, also with no significant intergroup differences (P > 0.05). Nearly all DNA samples exhibited purity within the accepted range of 1.8–2.1 (Ahlberg et al., 2021; Qian et al., 2017), indicating that high-purity DNA can be consistently extracted from different organ types using the same method.

### Yields and quality of DNA extracted from tissues of different organ types

DNA yields were compared across organ types. Concentrations were measured using the NanoDrop™ One, and yields were calculated based on the tissue weight used for extraction as previously studies did by normalizing yields to tissue weight (Hofstetter et al., 1997; Pearce et al., 2024; Austin et al., 2016; Stroh et al., 2021; Zeng et al., 2024; Siuta et al., 2023) (Figure 1B; Table 1). Yields were normalized to tissue weight by dividing the total amount of DNA (µg) by the tissue mass (mg) used for extraction. In C57BL/6J mice, the highest DNA yields were obtained from kidney (1.98 ± 0.33 μg/mg), spleen (2.03 ± 0.26 μg/mg), and colorectum (2.45 ± 0.61 μg/mg), while the lowest yields were from bone (0.22 ± 0.07 μg/mg) and muscle (0.38 ± 0.12 μg/mg). In BALB/c nude mice, the highest yields came from colorectum (4.50 ± 1.19 μg/mg), stomach (4.60 ± 0.69 μg/mg), and uterus (3.82 ± 0.71 μg/mg), and the lowest from bone (0.51 ± 0.13 μg/mg), heart (0.76 ± 0.14 μg/mg), and muscle (0.59 ± 0.05 μg/mg). Coefficients of variation (CV%) for DNA yields per organ type are presented in Table 4.

**TABLE 1:** The yields of DNA extracted from tissue of fourteen organ types.

Types of organs	C57BL/6J			BALB/c nude		
	Yield (ug/mg tissue)	Adjusted P (vs. colorectum)	Adjusted P (vs. bone)	Yield (ug/mg tissue)	Adjusted P (vs. colorectum)	Adjusted P (vs. bone)
Bone	0.22 ± 0.07	<0.0001	—	0.51 ± 0.13	<0.0001	—
Colorectum	2.45 ± 0.61	—	<0.0001	4.50 ± 1.19	—	<0.0001
Heart	0.58 ± 0.25	<0.0001	0.5025	0.76 ± 0.14	<0.0001	0.9976
Kidney	1.98 ± 0.33	0.2103	<0.0001	1.29 ± 0.23	<0.0001	0.3373
Liver	1.41 ± 0.21	<0.0001	<0.0001	3.09 ± 1.25	0.0086	<0.0001
Lung	1.69 ± 0.25	0.0061	<0.0001	1.76 ± 0.35	<0.0001	0.0259
Lymph node	1.10 ± 0.32	<0.0001	0.0009	NA	NA	NA
Muscle	0.38 ± 0.12	<0.0001	0.9951	0.59 ± 0.05	<0.0001	>0.9999
Ovary	0.62 ± 0.26	<0.0001	0.3728	1.39 ± 0.46	<0.0001	0.2121
Skin	0.50 ± 0.32	<0.0001	0.7876	3.42 ± 0.52	0.0739	<0.0001
Spleen	2.03 ± 0.26	0.3358	<0.0001	1.91 ± 0.53	<0.0001	0.0093
Stomach	1.03 ± 0.49	<0.0001	0.0025	4.60 ± 0.69	>0.9999	<0.0001
Thymus	1.75 ± 0.44	0.0153	<0.0001	NA	NA	NA
Uterus	0.62 ± 0.33	<0.0001	0.3834	3.82 ± 0.71	0.4976	<0.0001

Data were shown as mean ± standard deviation, n = 6.

NA: Not available.

Colorectum was selected as the reference tissue because it showed the highest DNA, yield in C57BL/6J mice, providing a consistent baseline for pairwise comparisons. For BALB/c nude mice, stomach showed similarly high yield, but colorectum was retained as reference for cross-strain consistency.

DNA quality was evaluated by gel electrophoresis (Figures 1C,D). Most extracts showed intact bands around 20 kb, though mild degradation was observed in DNA from colorectum, spleen, stomach, and thymus. These findings demonstrate that DNA yield varies by tissue type when using a standardized extraction protocol.

### Tissue quantity required for 10 μg DNA extraction

**FIGURE 2:**
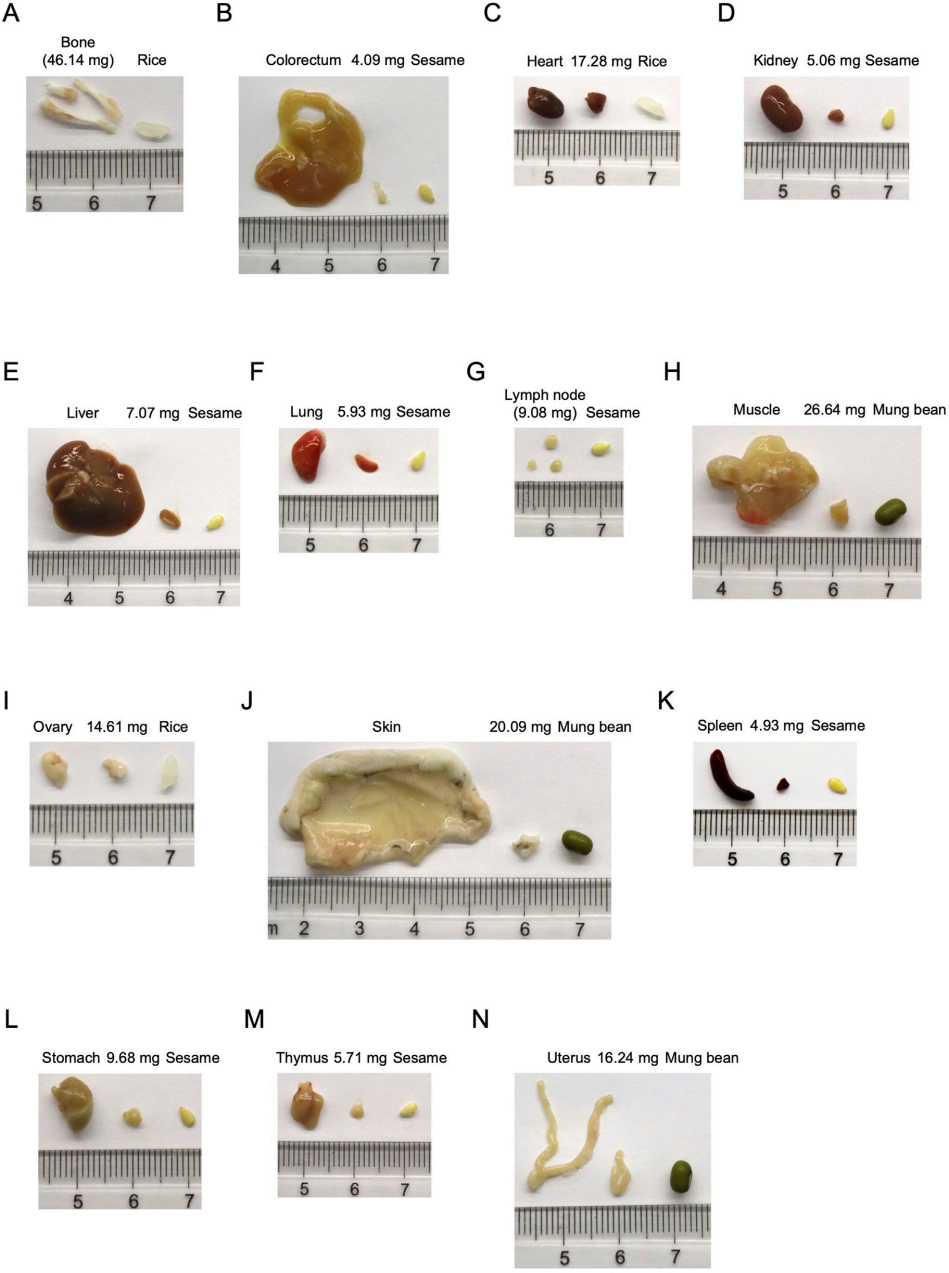
The amounts and sizes of tissue needed for the extraction of 10 μg of DNA in C57BL/6J mice. **(A-N)** Each figure presents the relative sizes of tissue for the extraction of 10 μg of DNA (middle), the corresponding intact organ (left, except for lymph node and bone; Muscle was obtained from both thighs; Skin was from the back of the mouse), and the referenced seed (right, sesame, rice, and mung bean).

To facilitate biobank sample handling, we determined the amount of tissue needed to obtain 10 μg of DNA, a typical requirement for applications such as PCR (While a single qPCR reaction requires only 10–100 ng of DNA, the total amount needed for a comprehensive experiment (including multiple targets, replicates, and standard curves) can reach 10–100 µg) and whole-genome sequencing (WGS). Correlation analysis confirmed a positive relationship between starting tissue weight and DNA yield within the same tissue type using the same extraction method (Supplementary Figure S1A). The tissue masses required to extract 10 μg DNA are then calculated and summarized in Table 5. Since weighing tissue manually is often impractical, we also provided visual references comparing tissue sizes to common seeds (e.g., sesame, rice, mung bean; Figure 2; Supplementary Figures S1B–M). For example, in C57BL/6J mice, 5.71 mg of thymus is comparable to a sesame seed, and 9.08 mg of lymph node tissue approximates three lymph nodes, each smaller than a sesame seed.

### Purity of RNA extracted from tissues of different organ types

**FIGURE 3:**
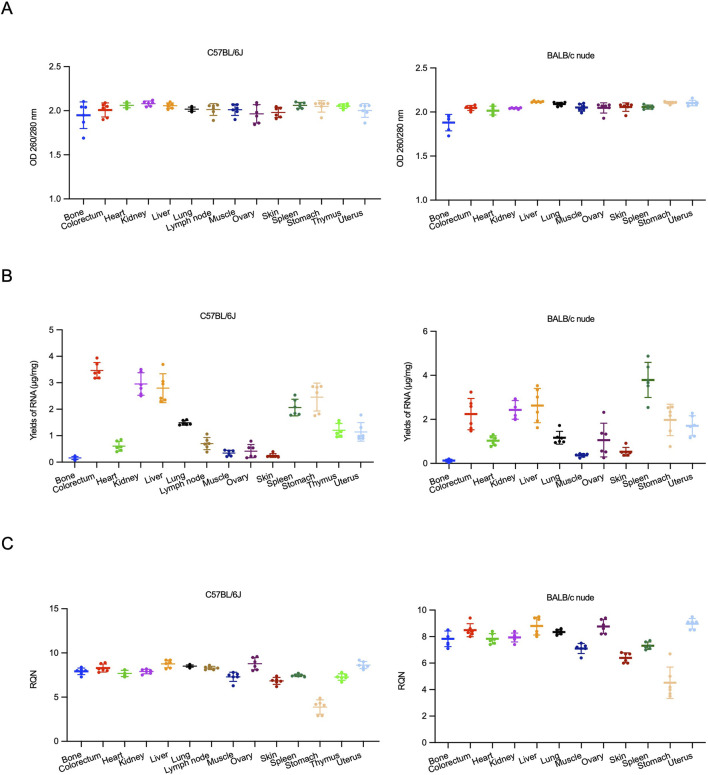
Purity, yields, and quality of RNA extracted from tissue of different types of organs. **(A)** The ratios of OD 260 nm to OD 280 nm of the RNA extracted from bone, colorectum, heart, kidney, liver, lung, lymph node, muscle, ovary, skin, spleen, stomach, thymus, and uterus in C57BL/6J (fourteen organ types) and BALB/c nude mice (twelve organ types). The results were shown as boxplots, n = 6. **(B)** Yields of the RNA extracted from tissue of twelve (BALB/c nude) to fourteen (C57BL/6J) organ types. The results were shown as boxplots (n = 6). **(C)** RNA integrity measured by capillary electrophoresis with Fragment Analyzer 5200. The results were shown as boxplots (n = 6). RQN: RNA Quality Number.

RNA was extracted from the same set of organ types. The 260/280 nm ratios were measured using a NanoDrop™ One (Figure 3A). In C57BL/6J mice, average ratios per group ranged from 1.95 ± 0.14 to 2.08 ± 0.03; in BALB/c nude mice, they ranged from 1.88 ± 0.09 to 2.10 ± 0.03. No significant differences were found among groups (P > 0.05). Nearly all values fell within the accepted purity range of 1.8–2.1 (Ahlberg et al., 2021; Qian et al., 2017), confirming that the extraction method consistently yields high-purity RNA across organ types.

### Yields and quality of RNA extracted from tissues of different organ types

RNA concentrations were measured, and yields were normalized to tissue weight by dividing the total amount of RNA (µg) by the tissue mass (mg) used for extraction (Figure 3B; Table 2). In C57BL/6J mice, the highest RNA yields were from colorectum (3.46 ± 0.28 μg/mg), kidney (2.96 ± 0.39 μg/mg), and liver (2.80 ± 0.50 μg/mg), while the lowest were from bone (0.16 ± 0.05 μg/mg), muscle (0.34 ± 0.10 μg/mg), and skin (0.25 ± 0.07 μg/mg). In BALB/c nude mice, the highest yields were from spleen (3.79 ± 0.73 μg/mg), liver (2.63 ± 0.71 μg/mg), and colorectum (2.24 ± 0.65 μg/mg), and the lowest from bone (0.13 ± 0.06 μg/mg), muscle (0.37 ± 0.07 μg/mg), and skin (0.53 ± 0.18 μg/mg). CV values are provided in Table 4.

**TABLE 2:** The yields of RNA extracted from tissue of fourteen organ types.

Types of organs	C57BL/6J			BALB/c nude		
	Yield (ug/mg tissue)	Adjusted P (vs. colorectum)	Adjusted P (vs. bone)	Yield (ug/mg tissue)	Adjusted P (vs. spleen)	Adjusted P (vs. bone)
Bone	0.16 ± 0.05	<0.0001	—	0.13 ± 0.06	<0.0001	—
Colorectum	3.46 ± 0.28	—	<0.0001	2.24 ± 0.65	<0.0001	<0.0001
Heart	0.60 ± 0.17	<0.0001	0.1139	1.03 ± 0.20	<0.0001	0.0388
Kidney	2.96 ± 0.39	0.0497	<0.0001	2.43 ± 0.39	0.0004	<0.0001
Liver	2.80 ± 0.50	0.0037	<0.0001	2.63 ± 0.71	0.0035	<0.0001
Lung	1.49 ± 0.08	<0.0001	<0.0001	1.17 ± 0.27	<0.0001	0.0119
Lymph node	0.71 ± 0.21	<0.0001	0.0273	NA	NA	NA
Muscle	0.34 ± 0.10	<0.0001	0.9413	0.37 ± 0.07	<0.0001	0.9874
Ovary	0.41 ± 0.23	<0.0001	0.7116	1.06 ± 0.70	<0.0001	0.0304
Skin	0.25 ± 0.07	<0.0001	0.9999	0.53 ± 0.18	<0.0001	0.7703
Spleen	2.07 ± 0.29	<0.0001	<0.0001	3.79 ± 0.73	—	<0.0001
Stomach	2.46 ± 0.48	<0.0001	<0.0001	1.98 ± 0.65	<0.0001	<0.0001
Thymus	1.21 ± 0.24	<0.0001	<0.0001	NA	NA	NA
Uterus	1.15 ± 0.32	<0.0001	<0.0001	1.71 ± 0.41	<0.0001	<0.0001

Data were shown as mean ± standard deviation, n = 6.

NA: Not available.

RNA quality was assessed by capillary electrophoresis (Figure 3C; Supplementary Figures S2, S3). The RNA quality number (RNA quality number (RQN)) ranged from 6 to 10 across all organ types except stomach, indicating good integrity. Thus, RNA yield also varies substantially across tissues under standardized extraction conditions.

### Tissue quantity required for 10 μg RNA extraction

**FIGURE 4:**
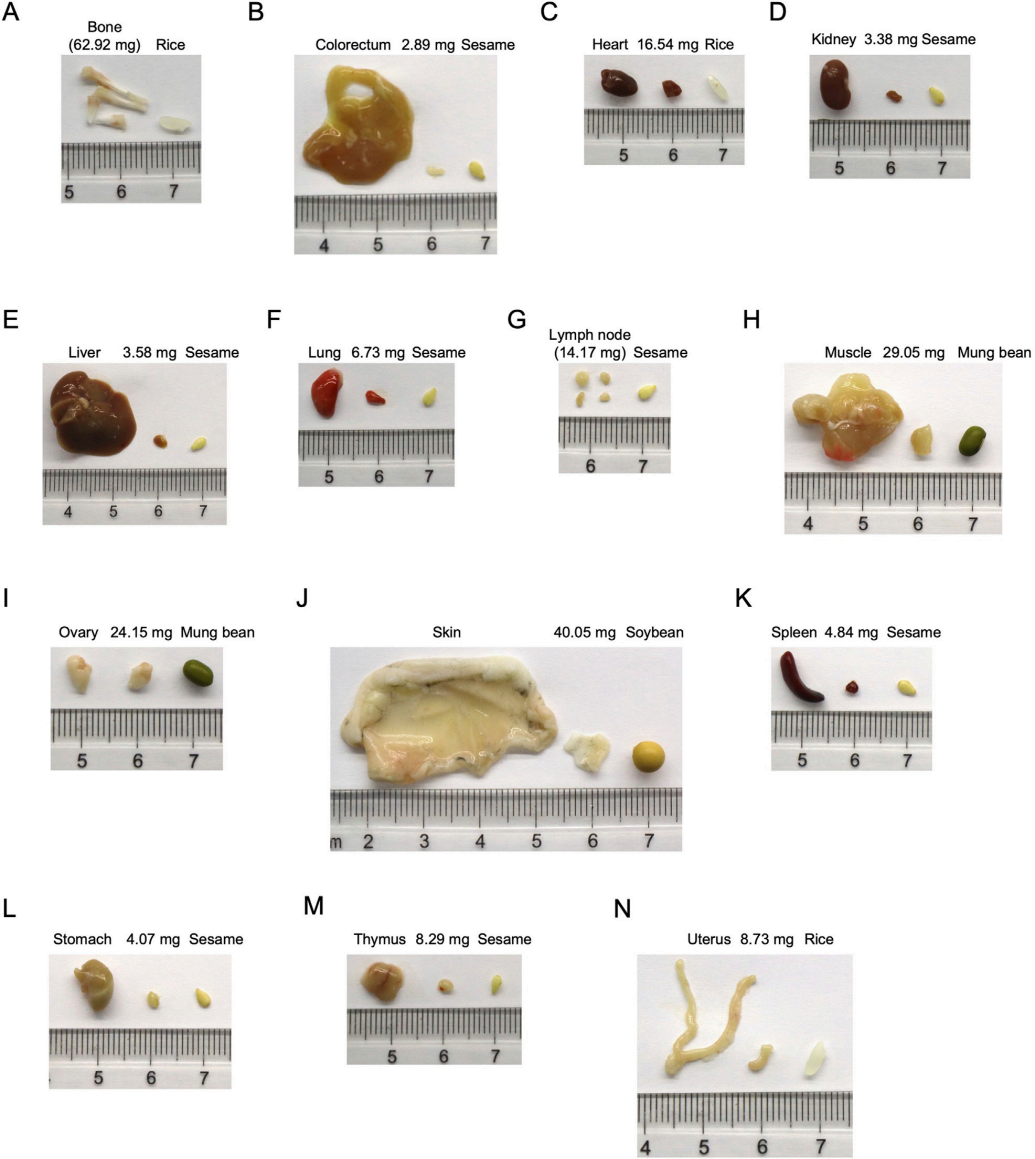
The amounts and sizes of tissue needed for the extraction of 10 μg of RNA in C57BL/6J mice. **(A-N)** Each figure presents the relative sizes of tissue for the extraction of 10 μg of RNA (middle), the corresponding intact organ (left, except for lymph node and bone. Muscle was obtained from both thighs. Skin was from the back of the mouse), and the referenced seed (right, sesame, rice, mung bean, and soybean).

We next determined the tissue mass required to obtain 10 μg of RNA, a common amount needed for real-time PCR or RNA-seq. A positive correlation was observed between tissue input and RNA yield within the same tissue group using the same extraction method (Supplementary Figure S4A). The tissue masses required to extract 10 μg RNA are then calculated and summarized in Table 5. Visual size comparisons using common seeds are provided in Figure 4 and Supplementary Figures S4B–M. For instance, in C57BL/6J mice, 6.73 mg of lung tissue is roughly equivalent to a sesame seed, and 14.17 mg of lymph node tissue corresponds to four lymph nodes, each smaller than a sesame seed.

### Yields of total protein extracted from tissues of different organ types

**FIGURE 5:**
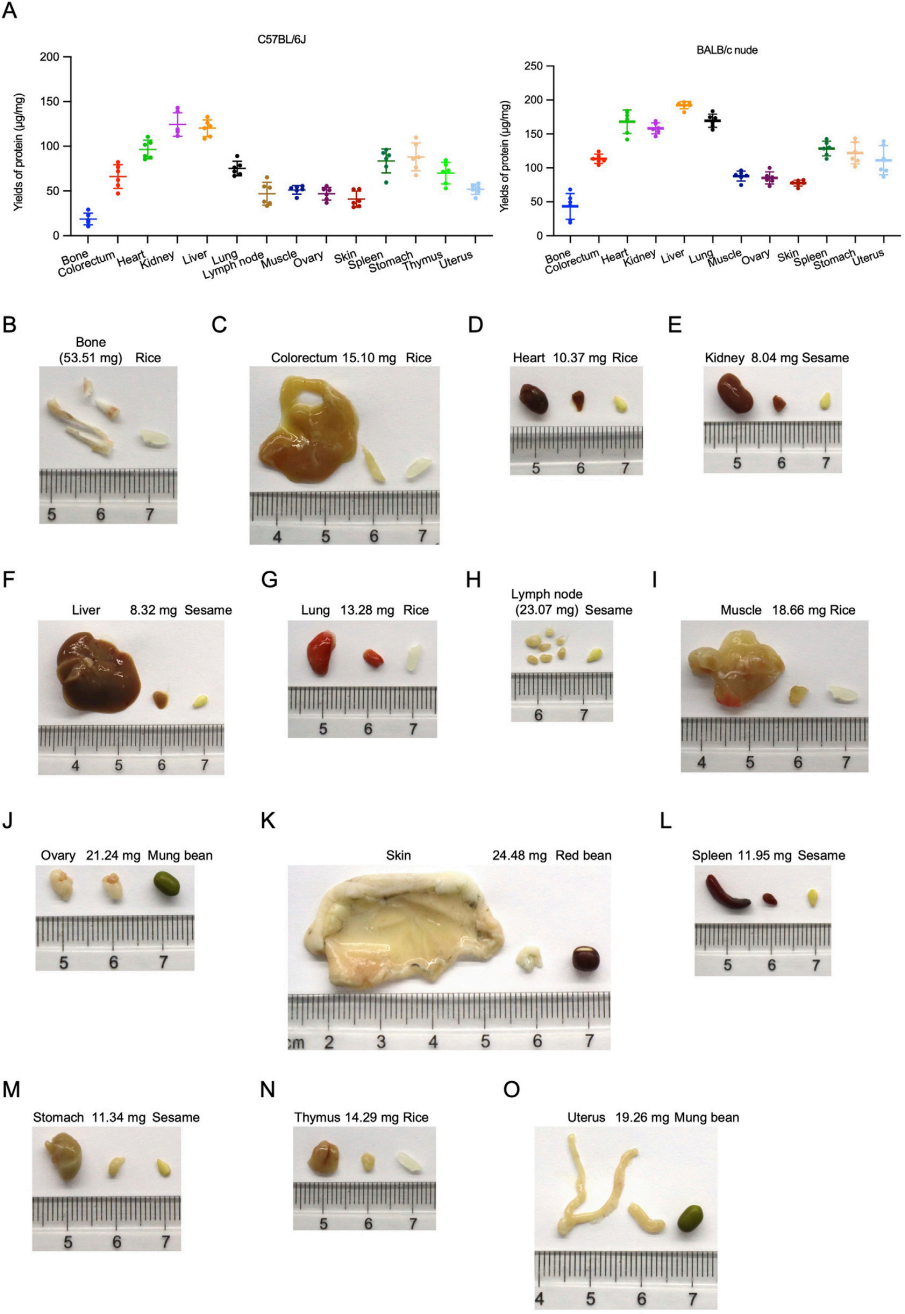
Yields of protein extracted from tissue of different types of organs. **(A)** Yields of the protein extracted from bone, colorectum, heart, kidney, liver, lung, lymph node, muscle, ovary, skin, spleen, stomach, thymus, and uterus in C57BL/6J (fourteen organ types) and BALB/c nude mice (twelve organ types). The results were shown as boxplots (n = 6). **(B–O)** Each figure presents the relative sizes of tissue for the extraction of 1 mg of protein (middle), the corresponding intact organ (left, except for lymph node and bone. Muscle was obtained from both thighs. Skin was from the back of the mouse), and the referenced seed (right, sesame, rice, mung bean, and red bean).

Total protein was extracted from all organ types using RIPA buffer (or a specialized kit for bone). Protein concentration was determined via BCA assay, and yields were normalized to tissue weight by dividing the total amount of protein (µg) by the tissue mass (mg) used for extraction. (Figure 5A; Table 3). In C57BL/6J mice, the highest protein yields were from kidney (124.39 ± 12.06 μg/mg) and liver (120.18 ± 8.44 μg/mg), and the lowest from bone (18.69 ± 5.91 μg/mg). In BALB/c nude mice, the highest yields were from liver (192.31 ± 4.90 μg/mg), lung (169.50 ± 8.83 μg/mg), and heart (168.10 ± 15.89 μg/mg), and the lowest from bone (43.34 ± 17.22 μg/mg). Coefficients of variation (CV%) for protein yields per organ type are presented in Table 4. These results indicate that protein yield is also highly tissue-dependent when using the same extraction method.

**TABLE 3:** The yields of protein extracted from tissue of fourteen organ types.

Types of organs	C57BL/6J			BALB/c nude		
	Yield (ug/mg tissue)	Adjusted P (vs. Kidney)	Adjusted P (vs. bone)	Yield (ug/mg tissue)	Adjusted P (vs. liver)	Adjusted P (vs. bone)
Bone	18.69 ± 5.91	<0.0001	—	43.34 ± 17.22	<0.0001	—
Colorectum	66.23 ± 12.1	<0.0001	<0.0001	113.31 ± 6.24	<0.0001	<0.0001
Heart	96.44 ± 9.50	0.0002	<0.0001	168.10 ± 15.89	0.0123	<0.0001
Kidney	124.39 ± 12.06	—	<0.0001	158.30 ± 7.27	0.0002	<0.0001
Liver	120.18 ± 8.44	0.9979	<0.0001	192.31 ± 4.90	—	<0.0001
Lung	75.33 ± 7.40	<0.0001	<0.0001	169.50 ± 8.83	0.0211	<0.0001
Lymph node	47.01 ± 11.71	<0.0001	0.0002	NA	NA	NA
Muscle	51.31 ± 4.38	<0.0001	<0.0001	87.96 ± 6.71	<0.0001	<0.0001
Ovary	47.09 ± 6.45	<0.0001	0.0002	85.19 ± 8.14	<0.0001	<0.0001
Skin	40.85 ± 8.28	<0.0001	0.0060	77.60 ± 3.98	<0.0001	0.0001
Spleen	83.65 ± 12.24	<0.0001	<0.0001	128.43 ± 9.72	<0.0001	<0.0001
Stomach	88.20 ± 14.25	<0.0001	<0.0001	121.89 ± 14.78	<0.0001	<0.0001
Thymus	69.96 ± 11.02	<0.0001	<0.0001	NA	NA	NA
Uterus	51.91 ± 5.28	<0.0001	<0.0001	111.22 ± 19.52	<0.0001	<0.0001

Data were shown as mean ± standard deviation, n = 6.

NA: Not available.

**TABLE 4:** The coefficients of variation (CV%) of the yields of DNA, RNA, and protein extracted from tissue of fourteen types of organs.

Coefficient of variation (%)	DNA		RNA		Protein	
	C57BL/6J	BALB/c nude	C57BL/6J	BALB/c nude	C57BL/6J	BALB/c nude
Bone	36.05	28.37	34.28	52.69	34.65	43.53
Colorectum	27.24	28.96	8.71	31.87	20.01	6.03
Heart	48.18	20.13	30.77	21.18	10.79	10.35
Kidney	18.02	19.45	14.32	17.40	10.62	5.03
Liver	16.31	44.33	19.52	29.71	7.69	2.79
Lung	16.12	21.80	5.83	24.95	10.76	5.71
Lymph node	32.34	NA	32.53	NA	27.30	NA
Muscle	34.69	9.86	31.09	21.20	9.36	8.35
Ovary	45.67	36.35	60.44	72.43	15.00	10.47
Skin	70.73	16.53	29.18	37.69	22.20	5.62
Spleen	14.06	30.50	15.30	21.02	16.03	8.29
Stomach	51.53	16.53	21.39	36.06	17.70	13.28
Thymus	27.42	NA	21.42	NA	17.26	NA
Uterus	57.85	20.46	30.50	26.6	11.15	19.22

NA: Not available.

### Tissue quantity required for 1 mg protein extraction

We calculated the amounts of tissue from various organ types required to extract a specific quantity of protein. We first conducted the correlation analysis using data from the same tissue group and the results indicated that the yields of protein exhibited a positive correlation with the weight of the starting material (Supplementary Figure S5A). Based on the results of correlation analysis, we calculated the amount of tissue needed to obtain 1 mg of protein (Table 5). Size comparisons are illustrated in Figures 5B–O and Supplementary Figures 5B–M. For example, in C57BL/6J mice, 14.29 mg of thymus is similar in size to a grain of rice, and 23.07 mg of lymph node tissue corresponds to seven lymph nodes, each smaller than a sesame seed.

**TABLE 5:** The amount of tissue (mg) required for the extraction of 10 µg of DNA, 10 µg of RNA, and 1 mg of protein.

Types of organs	The amount of tissue (mg) needed for the extraction of 10 μg of DNA		The amount of tissue (mg) needed for the extraction of 10 μg of RNA		The amount of tissue (mg) needed for the extraction of 1 mg of protein	
	C57BL/6J	BALB/c nude	C57BL/6J	BALB/c nude	C57BL/6J	BALB/c nude
Bone	46.14	19.68	62.92	77.90	53.51	23.07
Colorectum	4.09	2.22	2.89	4.47	15.10	8.83
Heart	17.28	13.08	16.54	9.70	10.37	5.95
Kidney	5.06	7.75	3.38	4.11	8.04	6.32
Liver	7.07	3.24	3.58	3.81	8.32	5.20
Lung	5.93	5.68	6.73	8.58	13.28	5.90
Lymph node	9.08	NA	14.17	NA	23.07	NA
Muscle	26.64	16.86	29.05	27.12	18.66	11.37
Ovary	14.61	7.17	24.15	9.43	21.24	11.74
Skin	20.09	2.93	40.05	18.85	24.48	12.89
Spleen	4.93	5.24	4.84	2.64	11.95	7.79
Stomach	9.68	2.17	4.07	5.06	11.34	8.20
Thymus	5.71	NA	8.29	NA	14.29	NA
Uterus	16.24	2.62	8.73	5.86	19.26	8.99

NA, Not available.

### Correlations among DNA, RNA, and protein yields between the two mouse strains

**FIGURE 6:**
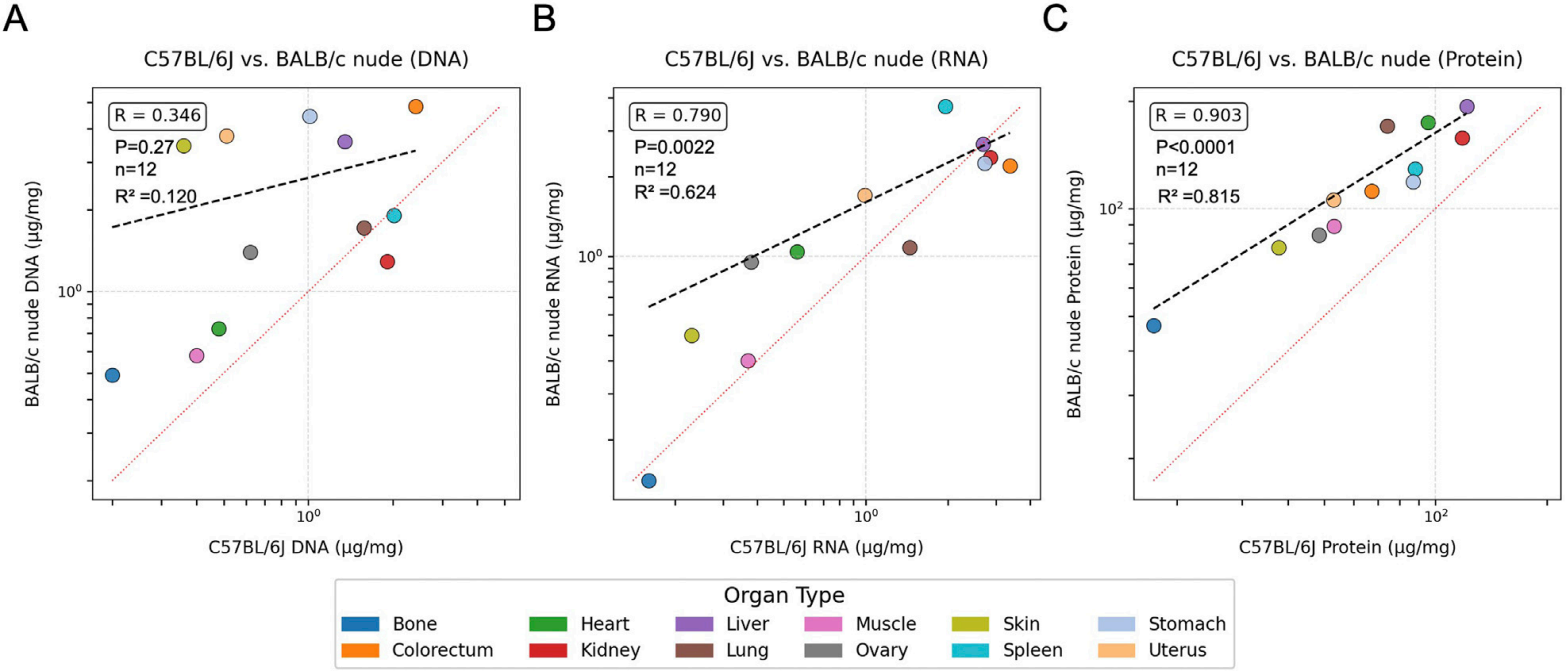
The correlation between the DNA **(A)**, RNA **(B)**, and protein **(C)** yields of C57BL/6 mice with that of BALB/c nude mice. Linear regression was performed using GraphPad Prism 9.3.0, with yield per tissue type as individual data points.

Finally, we analyzed the correlation between DNA, RNA, and protein yields of C57BL/6J mice and BALB/c nude mice based on the weight-normalized median data points per organ (Figures 6A–C). The results showed a weak (DNA) or strong (RNA and protein) positive correlation in the yields between the two strains, suggesting similarity at least in the RNA and protein yields of the two strains.

## Discussion

In this study, we analyzed the yields of DNA, RNA, and protein extracted from various mouse tissue types, establishing a general baseline for these biomolecules across fourteen organ types using well-established extraction methods, specifically, widely available commercial kits and standard protocols for nucleic acid and protein isolation. Our results indicated that DNA yields were higher in organ types such as the colorectum and lower in bone. Similarly, RNA yields were elevated in the colorectum and spleen but reduced in bone. Protein yields were notably higher in the liver and kidney, while lower levels were detected in bone.

Based on these findings, we can estimate the amount of tissue from C57BL/6J and BALB/c nude mice required for downstream analyses. We calculated the tissue quantities needed to obtain specific amounts of DNA, RNA, and protein, such as 10 μg of DNA, 10 μg of RNA, and 1 mg of protein, from both mouse strains. The corresponding physical sizes of these tissue amounts were illustrated using common seeds like sesame and rice as reference, aiding in practical estimation of sample requirements.

This baseline information can also guide the storage of mouse tissue samples in biobanks. For organ types yielding higher amounts of DNA, RNA, and protein, both storage volume and physical size can be reduced. Conversely, for organ types with lower biomolecule yields, larger storage volumes and sample sizes are recommended. Our systematic analysis of extraction yields across murine tissues offers actionable insights to improve biobanking strategies. These yield metrics support evidence-based allocation of limited tissue resources, especially for small organ types. By defining tissue-specific minimum masses required for reliable extraction of each biomolecule, our data help minimize waste while ensuring adequate material for downstream applications.

It is important to note that several factors may influence nucleic acid and protein yields, including the physiological state of the tissue (e.g., healthy vs. diseased), extraction methodologies (including homogenization techniques), and storage conditions (e.g., cryopreservation vs. paraffin-embedding) and duration. Therefore, the conclusions of this study are most directly applicable to experimental systems and mouse strains similar to those used here. Nonetheless, our data may still serve as a useful reference in other contexts. For example, in disease models where necrosis is present, larger tissue amounts may be needed for extraction or storage. Additionally, fixatives such as formalin and Bouin’s solution can adversely affect the yield and quality of DNA, RNA, and protein. Previous studies have shown that cryopreserved tissues generally yield more DNA than paraffin-embedded samples, and prolonged storage of paraffin sections leads to increased degradation of nucleic acids (Okojie et al., 2024; Baloglu et al., 2008; Yi et al., 2020). Hence, under such conditions, larger tissue samples would be required. Conversely, if more efficient extraction methods are used, such as the TRIzol™ method for RNA (Ban et al., 2013; Sultan et al., 2014), smaller initial tissue amounts or storage volumes may be sufficient.

This study has several limitations. The extraction methods employed here are commonly used in contemporary biomedical research and are suitable for various downstream applications, including PCR, WGS, and whole-genome bisulfite sequencing (WGBS). Future studies could benefit from including data derived from alternative extraction protocols. Secondly, based on our quality assessments, mild degradation was observed in DNA or RNA extracted from the colorectum, spleen, stomach, and thymus. Given the high nuclease activity in these tissues, incorporating nuclease inhibitors (e.g., RNAlater) or rapid freezing in liquid nitrogen is recommended to prevent biomolecule degradation. Thus, for freshly collected tissues intended for direct analysis, implementing such pretreatment steps should be considered in future workflows. Additionally, the sample size (six mice per group) is relatively small and may not fully capture biological variability. Certain tissue types (e.g., bone, skin) exhibited relatively high coefficients of variation (%CV) for DNA, RNA, or protein yields. This variability may stem from both biological and technical factors. Biologically, tissues such as bone and skin are inherently heterogeneous, containing variable proportions of mineralized matrix, connective tissue, and cellular components, which can lead to substantial inter-individual differences in extractable biomolecules. Technically, complete homogenization of these tough tissues is more challenging, and residual nuclease or protease activity may differ between samples. These factors likely contribute to the observed higher %CV values. Future studies with larger sample sizes and optimized tissue-processing protocols may help further reduce this variability. Finally, the elution volumes of 50 µL for DNA and 40 µL for RNA were selected to achieve sufficient concentration for downstream applications while minimizing sample dilution. These volumes are within the ranges recommended by the kit manufacturers (QIAamp DNA Mini Kit: 50–200 μL; RNEasy Plus Universal Mini Kit: 30–50 µL). It should be noted that alternative elution volumes may affect yield and concentration.

## Conclusion

The rational and efficient preservation and management of biological samples are essential for supporting high-quality scientific research. In this study, we extracted DNA, RNA, and protein from multiple mouse organ types and compared the yields of these biomolecules across different tissue types. We established a general baseline for the extraction of DNA, RNA, and protein from fourteen types of mouse tissues, thereby providing a valuable foundation for the economical and efficient preservation of mouse tissue samples.

## Data Availability

The raw data supporting the conclusions of this article will be made available by the authors, without undue reservation.

## Supplementary material

SUPPLEMENTARY FIGURE 1
**(A)** The correlation between the yield of DNA with the weight of the starting material from the bone tissue of BALB/c nude mice. **(B–M)** The amounts and sizes of tissue needed for the extraction of 10 μg of DNA in BALB/c nude mice. Each figure presents the relative sizes of tissue for the extraction of 10 μg of DNA (middle), the corresponding intact organ (left, except for bone. Muscle was obtained from both thighs. Skin was from the back of the mouse), and the referenced seed (right, sesame, rice, and mung bean).

SUPPLEMENTARY FIGURE 2 Exemplary electropherograms of RNA integrity measured by capillary electrophoresis with Fragment Analyzer 5200 in C57BL/6J mice.

SUPPLEMENTARY FIGURE 3 Exemplary electropherograms of RNA integrity measured by capillary electrophoresis with Fragment Analyzer 5200 in BALB/c nude mice.

SUPPLEMENTARY FIGURE 4
**(A)** The correlation between the yield of RNA with the weight of the starting material from the heart tissue of BALB/c nude mice. **(B–M)** The amounts and sizes of tissue needed for the extraction of 10 μg of RNA in BALB/c nude mice. Each figure presents the relative sizes of tissue for the extraction of 10 μg of RNA (middle), the corresponding intact organ (left, except for bone. Muscle was obtained from both thighs. Skin was from the back of the mouse), and the referenced seed (right, sesame, rice, and mung bean).

SUPPLEMENTARY FIGURE 5
**(A)** The correlation between the yield of protein with the weight of the starting material from the lymph node tissue of C57BL/6J mice. **(B–M)** The amounts and sizes of tissue needed for the extraction of 1 mg of protein in BALB/c nude mice. Each figure presents the relative sizes of tissue for the extraction of 1 mg of protein (middle), the corresponding intact organ (left, except for bone. Muscle was obtained from both thighs. Skin was from the back of the mouse), and the referenced seed (right, sesame and rice).
