# Supplementary material for: The yields of nucleic acids and proteins extracted from various murine tissue types
Source: Acta Biochim Pol. 2026 Jul 29;73:16122. doi: 10.3389/abp.2026.16122 (PMC13461566; doi:10.3389/abp.2026.16122)

Supplementary Figure 1

A

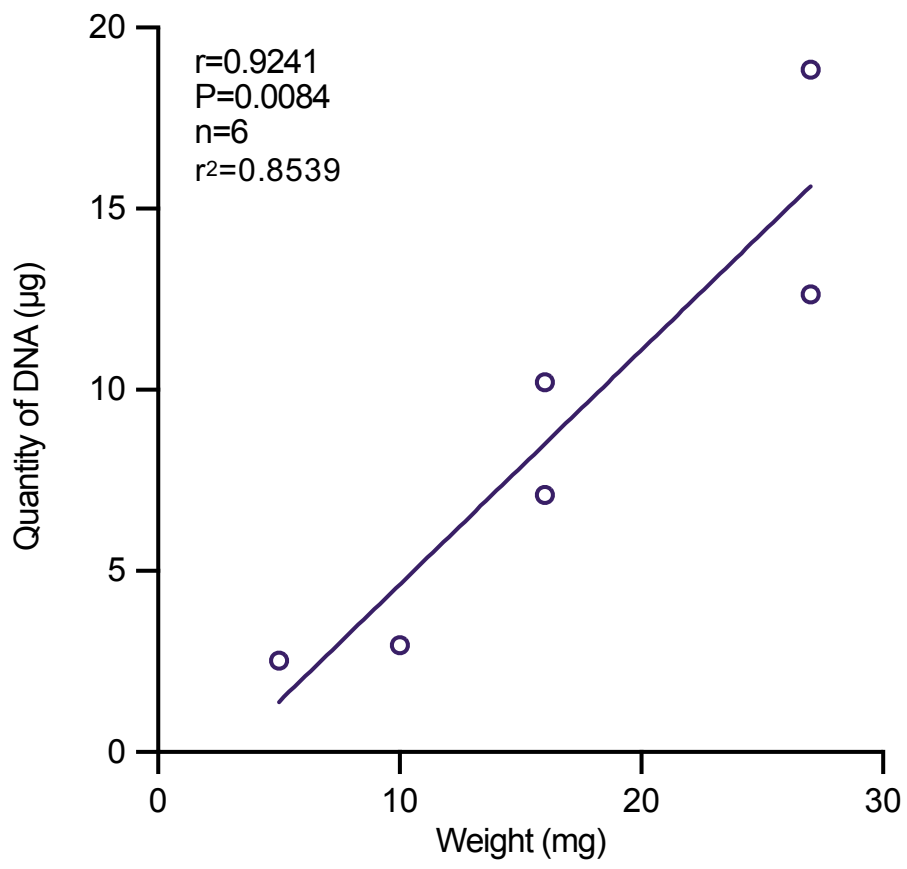

B

Bone (19.68 mg) Rice

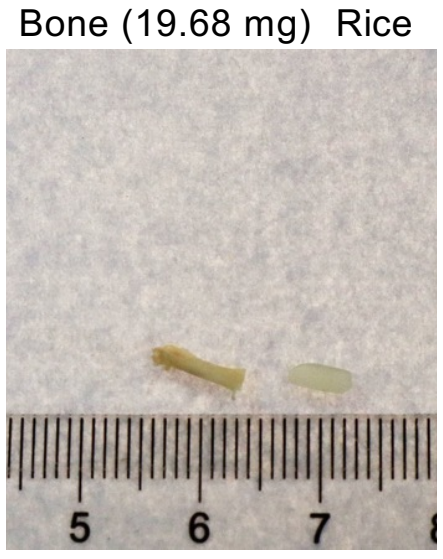

C

Colorectum 2.22 mg Sesame

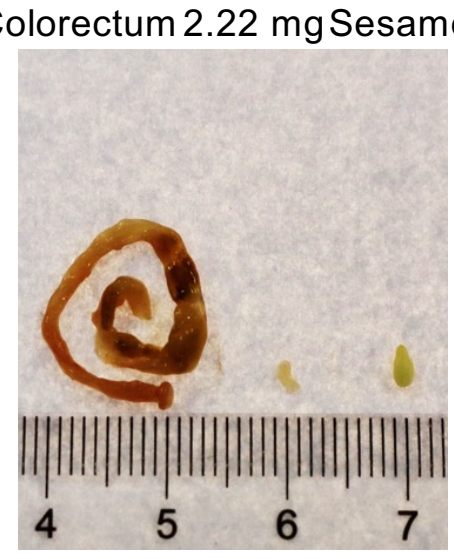

D

Heart 13.08 mg Rice

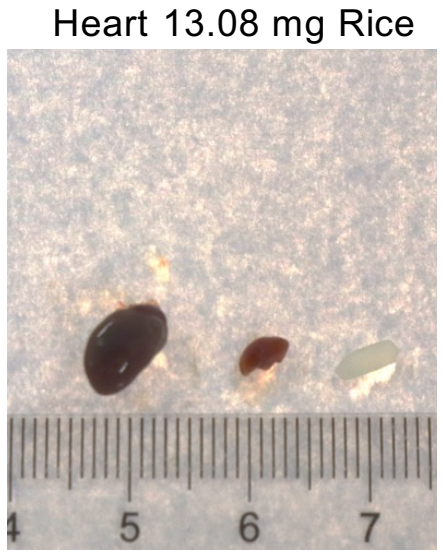

E

Kidney 7.75 mg Sesame

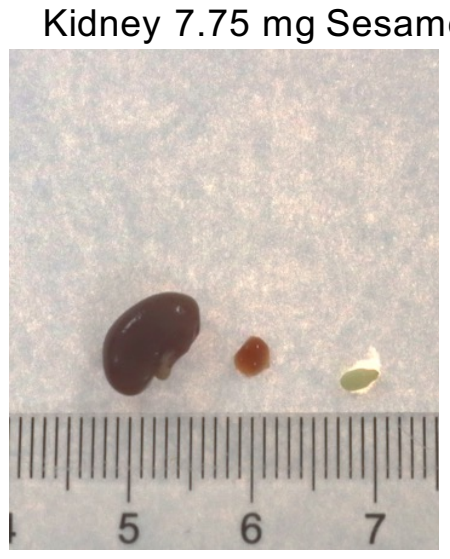

F

Liver 3.24 mg Sesame

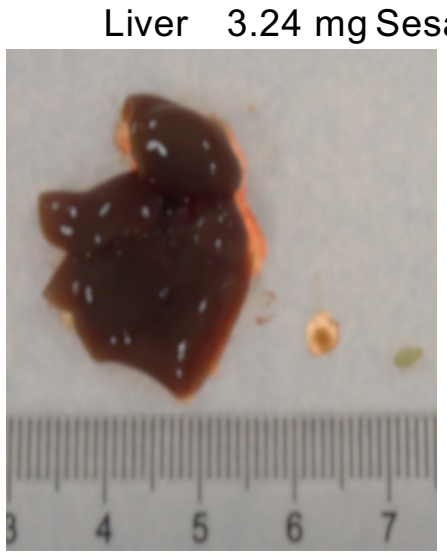

G

Lung 5.68 mg Sesame

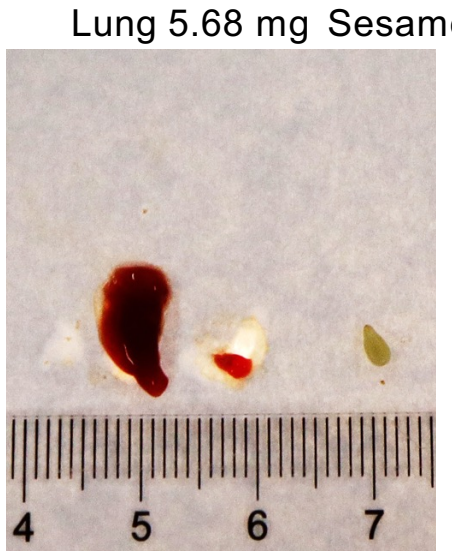

H

Muscle 16.86 mg Mung bean

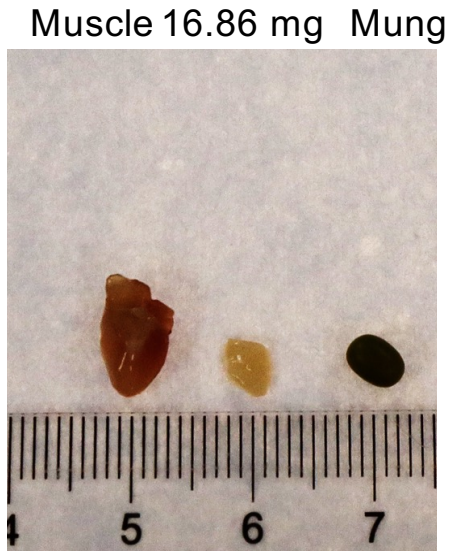

I

Ovary 7.17 mg Sesame

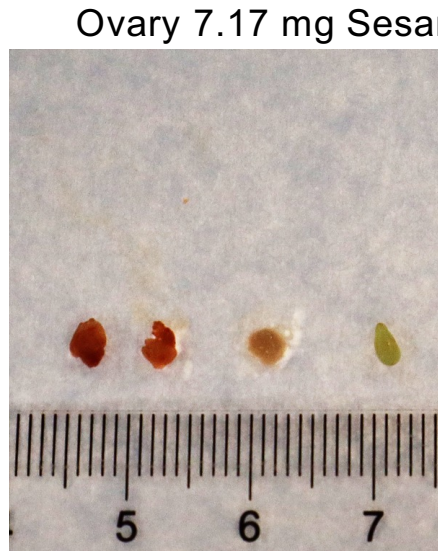

J

Skin 2.93 mg Sesame

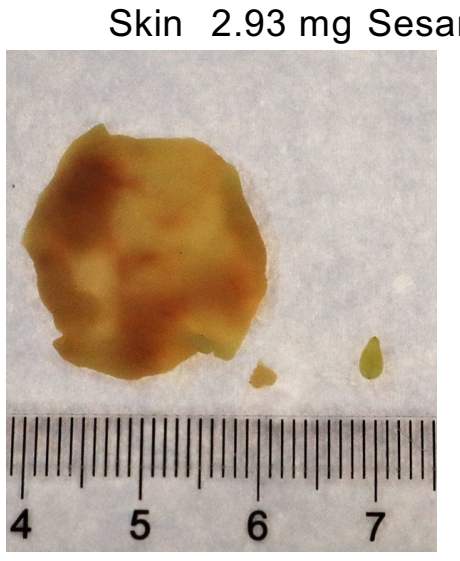

K

Spleen 5.24 mg Sesame

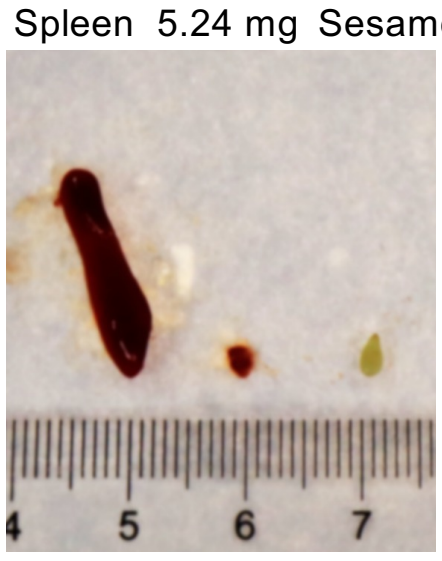

L

Stomach 2.17 mg Sesame

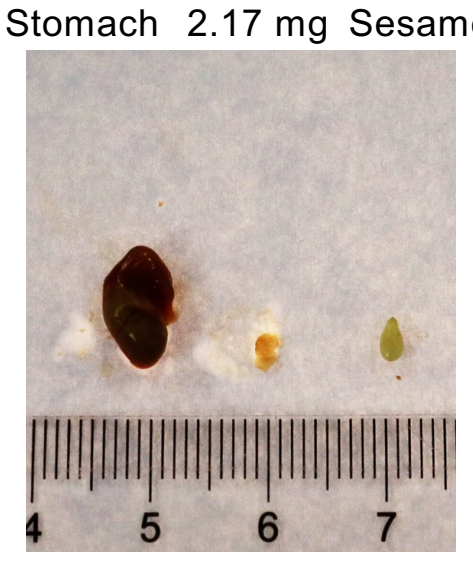

M

Uterus 2.62 mg Sesame

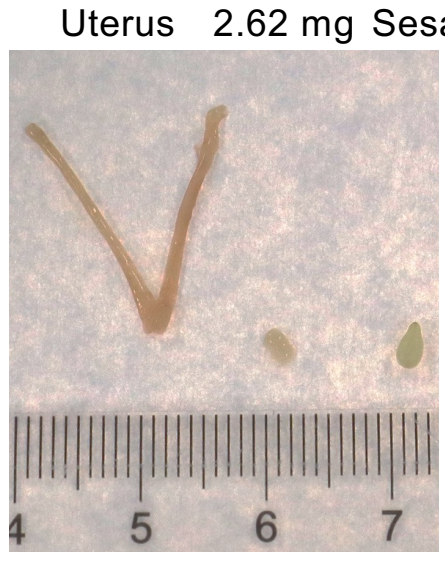

Supplementary Figure 2

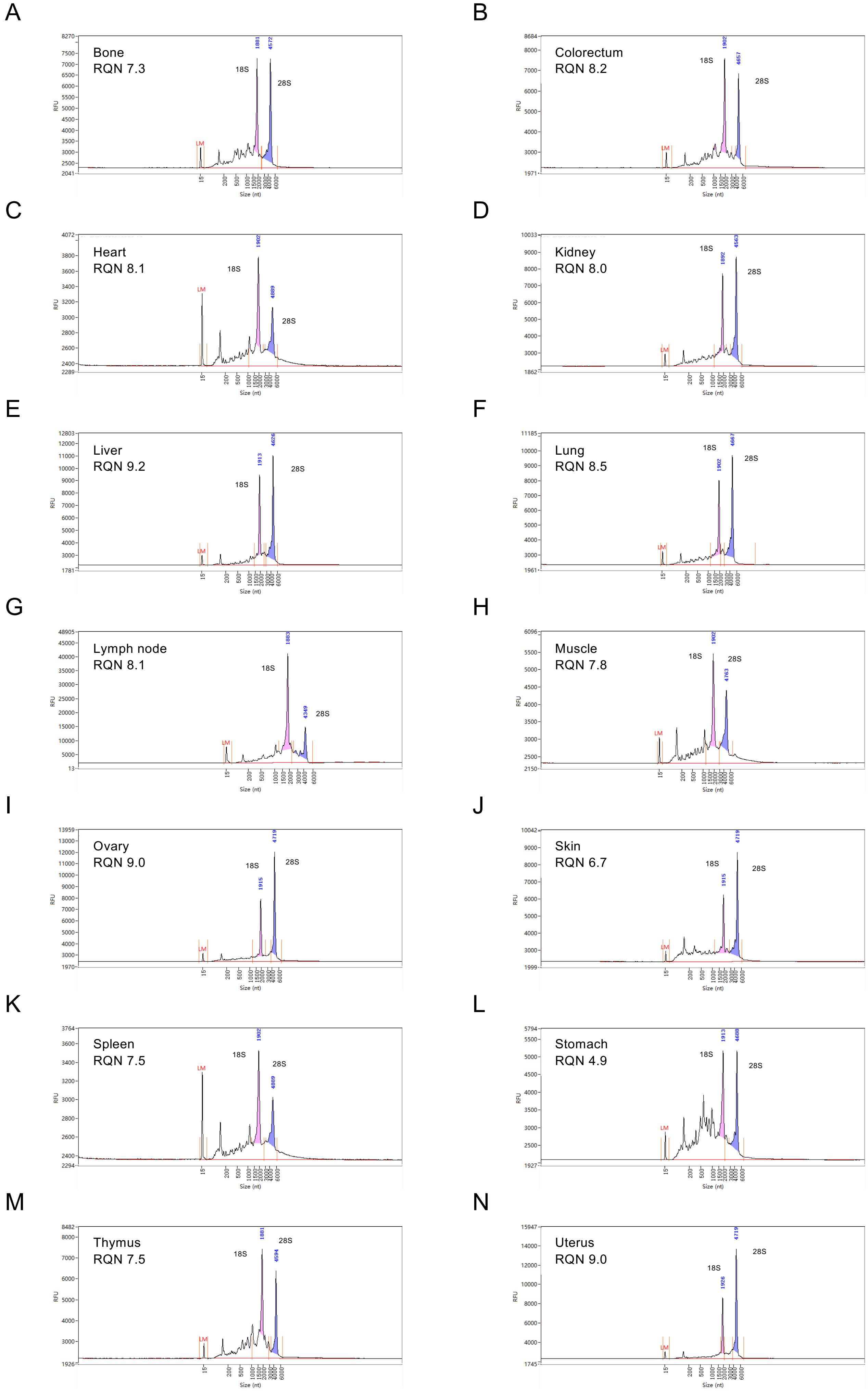

Supplementary Figure 3

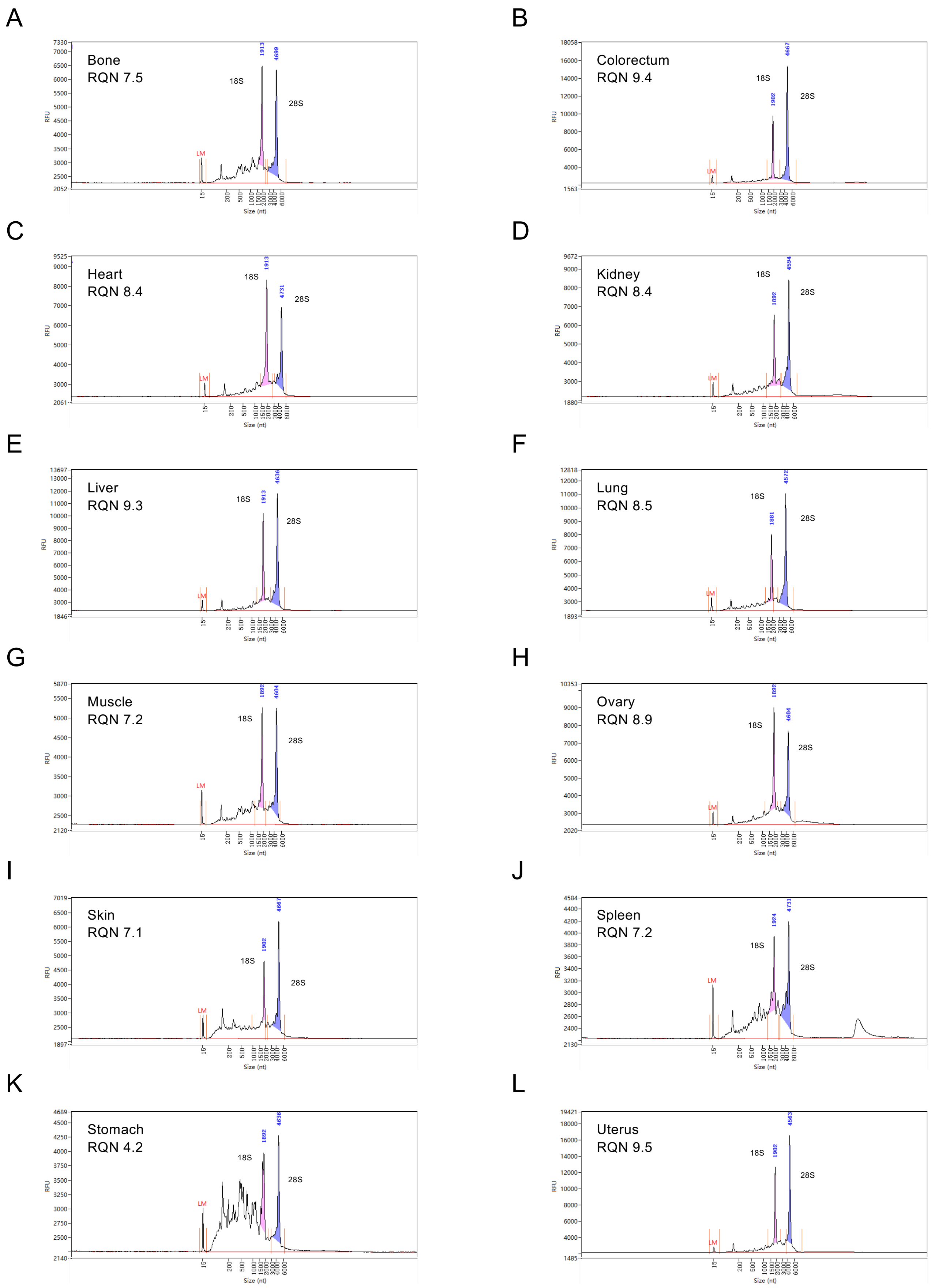

Supplementary Figure 4

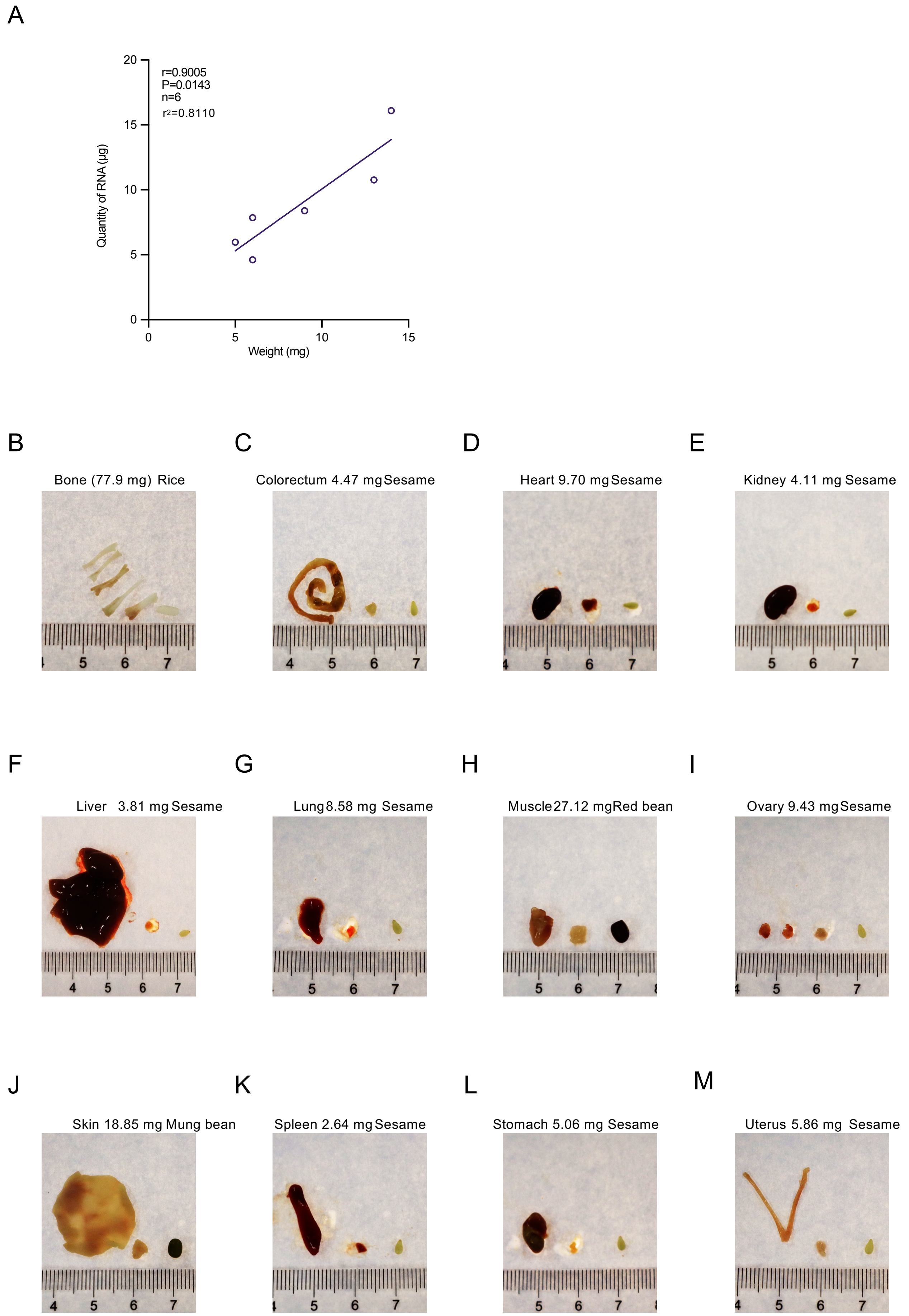

Supplementary Figure 5

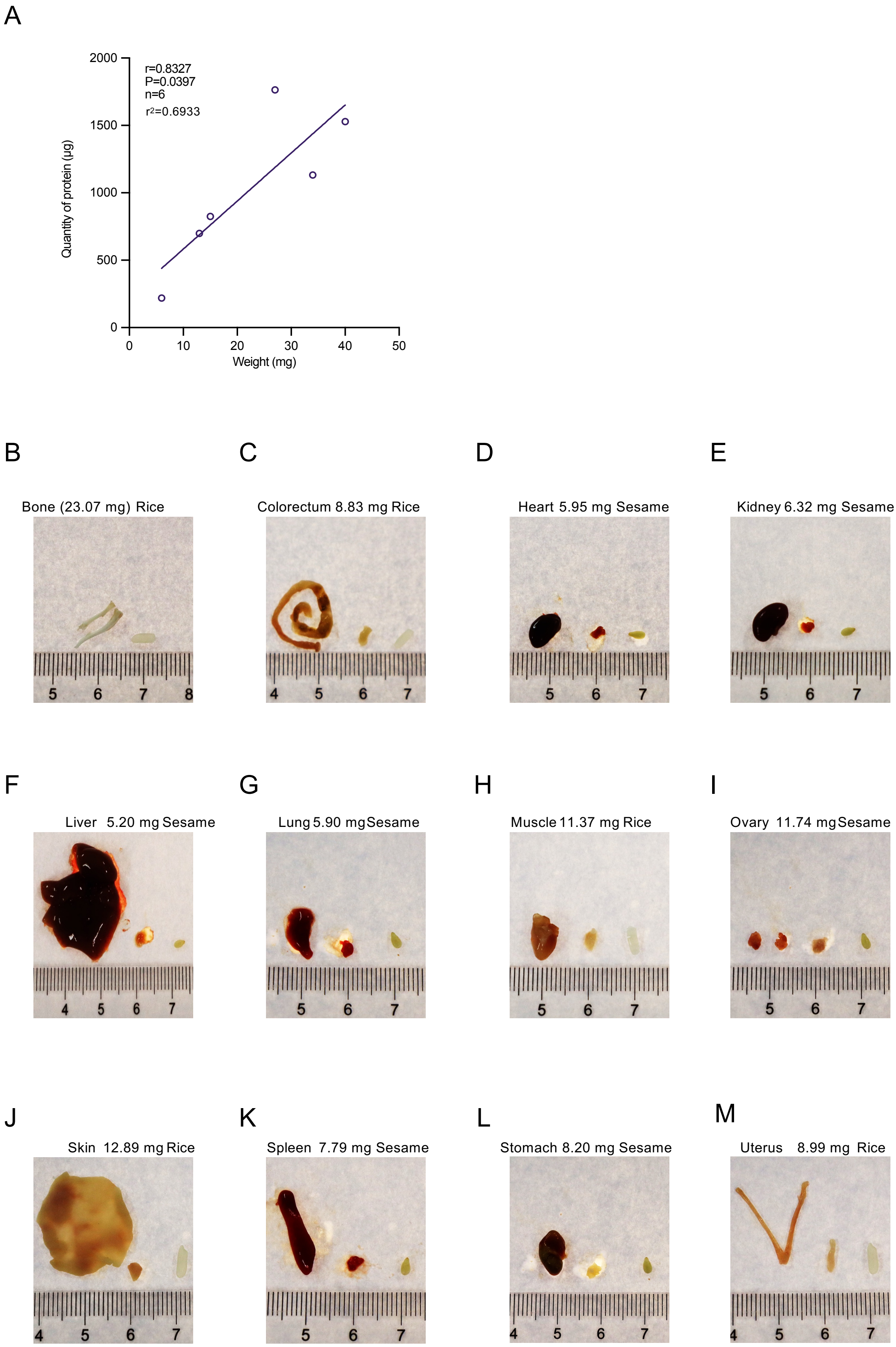

Supplement: Supplementary file 1 [file DataSheet1.pdf]
